# Supplementary material for: Content-rich biological network constructed by mining PubMed abstracts
Source: BMC Bioinformatics. 2004 Oct 8;5:147. doi: 10.1186/1471-2105-5-147 (PMC528731; doi:10.1186/1471-2105-5-147)
Supplement: Additional File 2 — The original results of the above study (non-essential files are deleted to keep the file size under the limit set by BMC bioinformatics). [file 1471-2105-5-147-S2.bz2 › chilibotAdditionalFile2/dip50/162ID8612709E567/html/ACVR1B_ACVR2.html]

 


 **ACVR1B** and **ACVR2** 
  
Found 46 abstracts in PubMed, retrieved 46.  
 

 What does Google say? 
 PDF only 
| .edu only 

---

**Interactive relationship** (e.g. stimulation, inhibition, etc)

**Neutral relationship**- Isoform analysis of the major functional type I activin receptor  [ **ACVR2** ]  alk4  [ **ACVR1B** ]  revealed the full length transcript SKR2  [ **ACVR1B** ]  1 and transcripts for the partially truncated proteins SKR2  [ **ACVR1B** ]  2 and SKR2  [ **ACVR1B** ]  3 in normal thyroids and goiters.  Ref: 11069203 Horm Metab Res, 2000
- This strongly suggests that B1 ALK 4  [ **ACVR1B** ]  ActR IB  [ **ACVR1B** ]  could be the type I activin receptor  [ **ACVR2** ] , as type I and type II activin receptor  [ **ACVR2** ]  were supposed to form a receptor complex.  Ref: 9013782 Brain Res Mol Brain Res, 1996
- Here we show that beta beta activin binds to the activin type II receptor kinase ActRII  [ **ACVR2** ]  which induces activin binding to the type I receptor kinase SKR2  [ **ACVR1B** ]  to form ActRII  [ **ACVR2** ] .beta beta.SKR2  [ **ACVR1B** ]  complexes in which an activin beta chain occupies each receptor subunit.  Ref: 7890768 J Biol Chem, 1995
- CONCLUSIONS These results suggest that activin A binds to a receptor complex of ActR II  [ **ACVR2** ]  and ActR IB  [ **ACVR1B** ] , and preferentially activates Smad3 in HaCaT human keratinocytes.  Ref: 9605406 Genes Cells, 1998
- Activins bind specific type II receptor serine kinases ActRII  [ **ACVR2** ]  or IIB to promote the recruitment and phosphorylation of the type I receptor serine kinase, ALK4  [ **ACVR1B** ]  refs 7 9, which then regulates gene expression by activating Smad proteins.  Ref: 10746731 Nature, 2000
- Each of the alanine substituted ActRII  [ **ACVR2** ]  mutants retaining activin binding maintains the ability to form cross linked complexes with activin and supports activin cross linking to the type I activin receptor  [ **ACVR2** ]  ALK4  [ **ACVR1B** ] .  Ref: 10652306 J Biol Chem, 2000
- At E15, the expression of ActR II  [ **ACVR2** ]  mRNA was stronger than that of BMPR II in the spinal cord, followed in intensity by the expression of ActR I, ActR IB  [ **ACVR1B** ] , BMPR IA, BMPR IB and TbetaR I mRNA.  Ref: 8854897 Cell Tissue Res, 1996
- A kinase deleted version of ALK4  [ **ACVR1B** ]  can form an inactive complex with activin and ActRII  [ **ACVR2** ]  IIB and thereby acts in a dominant negative manner to block activin signaling.  Ref: 12665502 J Biol Chem< MedlineTA>J Biol Chem, 1996

**Non-interactive relationship** (e.g. studied together, co-existance, homology, etc.)

- The evidence suggests that ActR IIB acts as a primary activin receptor  [ **ACVR2** ]  and ActR IB  [ **ACVR1B** ]  acts as a downstream transducer of activin signals.  Ref: 8622651 Mol Cell Biol, 1996
- Activin A bound to ActR IB  [ **ACVR1B** ]  ALK 4  [ **ACVR1B** ]  and ActR II  [ **ACVR2** ] , and BMP 7 bound to ActR I ALK 2, BMP type I receptor BMPR I ALK 3, ActR II  [ **ACVR2** ]  and BMPR II.  Ref: 10574705 J Cell Sci, 1999
- Both type I and type II activin receptor  [ **ACVR2** ]  ActR I, ActR IB  [ **ACVR1B** ]  and ActR II  [ **ACVR2** ]  mRNA expression was also detectable in the pheochromocytoma tissues.  Ref: 10810314 J Endocrinol, 2000
- The Activin receptor  [ **ACVR2** ]  ALK4  [ **ACVR1B** ]  is also able to mediate Nodal signaling but only in the presence of Cripto, with which it can also interact directly.  Ref: 11485994 Genes Dev, 2001
- Expression of dominant negative mutants of various receptors indicated that ALK4  [ **ACVR1B** ] , and either ActRIIA  [ **ACVR2** ]  or ActRIIB, function as type I and type II receptors for Nodal, respectively.  Ref: 11952836 Genes Cells, 2002
- The type I receptors examined were the TGF beta type I receptor TbetaR I, activin type I receptor ActR I, activin type IB receptor ActR IB  [ **ACVR1B** ] , bone BMP type IA receptor BMPR IA, BMP type IB receptor BMPR IB, and activin receptor  [ **ACVR2** ]  like kinase 1 ALK 1.  Ref: 10321529 Acta Ophthalmol Scand, 1999
- The orphan receptor ALK7 and the Activin receptor  [ **ACVR2** ]  ALK4  [ **ACVR1B** ]  mediate signaling by Nodal proteins during vertebrate development.  Ref: 11485994 Genes Dev, 2001
- Crosslinking studies measuring binding of 125I activin A to the ALK4  [ **ACVR1B** ]  trunc mutants in the presence of ActRII  [ **ACVR2** ]  implicated the same residues.  Ref: 12665502 J Biol Chem< MedlineTA>J Biol Chem, 2001
- Molecular cloning has yielded genes encoding two type II receptors ActRII  [ **ACVR2** ]  and ActRIIB and at least two type I receptors ALK2 and ALK4  [ **ACVR1B** ] .  Ref: 8971337 Biochem Soc Symp, 1996
- RESULTS Activin A and receptors ActR I, ActR IB  [ **ACVR1B** ] , ActR II  [ **ACVR2** ]  are transcribed in corneal fibroblasts.  Ref: 11773015 Invest Ophthalmol Vis Sci, 2002
- To explore the molecular cascade of odontoblast differentiation, Northern blot analyses and reverse transcriptase polymerase chain reaction were here used to investigate the expression patterns of the genes for TGF beta superfamily members TGF beta 1, namely bone morphogenetic protein BMP 4, BMP 7, activin beta A and activin beta B, and their type I and type II receptors, namely activin receptor  [ **ACVR2** ]  like kinase ALK 2 ActR I, ALK 3 BMPR IA, ALK 4  [ **ACVR1B** ]  ActR IB  [ **ACVR1B** ] , ALK 5 T beta R I, BMPR II and T beta R II, during differentiation of pulp cells into preodontoblasts in bovine adult pulp cell culture.  Ref: 9296267 Arch Oral Biol, 1997
- This effect was mediated, at least in part, by the type IB activin receptor  [ **ACVR2** ]  ALK4  [ **ACVR1B** ] .  Ref: 11034308 FEBS Lett, 2000
- The presence of inhibin subunits alpha, betaA and betaB, activin binding protein follistatin and activin receptors ActR I, ActR IB  [ **ACVR1B** ] , ActR II  [ **ACVR2** ]  and ActR IIB mRNA transcripts in bovine oocytes and preimplantation embryos was examined by reverse transcription polymerase chain reaction.  Ref: 11596877 Reprod Fertil Dev, 1998
- The recently cloned bone morphogenetic factor receptor type II BMPR II was compared with the ActR II  [ **ACVR2** ]  and several type I receptors including ActR I, ActR IB  [ **ACVR1B** ] , BMPR IA, BMPR IB and TbetaR I.  Ref: 8854897 Cell Tissue Res, 1996
- Primers specific for two type I and two type II activin receptors ActR I, ActR IB  [ **ACVR1B** ] , ActR II  [ **ACVR2** ] , and ActR IIB were used in polymerase chain reaction PCR to amplify cDNAs prepared from granulosa luteal cells, placental tissues and isolated trophoblast cells.  Ref: 10210281 Life Sci, 1999
- The expression of both type I ALK 4  [ **ACVR1B** ]  ActRIB and type II ActRII  [ **ACVR2** ]  receptors for activin was detected in F5 5.fl cells.  Ref: 11078996 Eur J Endocrinol, 2000
- The effect of activin is most likely mediated through specific receptors as mRNAs encoding several forms of activin receptors, namely ActR I, ActR IB  [ **ACVR1B** ] , ActR II  [ **ACVR2** ]  and ActR IIB are found in the preovulatory follicles as well as in cultured granulosa luteal cells.  Ref: 8836491 Biol Signals< MedlineTA>Biol Signals, 2000
- ActR II  [ **ACVR2** ] , ActR I, ActR IB  [ **ACVR1B** ]  and, weakly, TbetaR I were all expressed in the dentate gyrus.  Ref: 8854897 Cell Tissue Res, 1996
- Inhibin also binds to ActRII  [ **ACVR2** ]  through its beta subunit, competes with the binding of activin to ActRII  [ **ACVR2** ] , but fails to form the ActRII  [ **ACVR2** ] .SKR2  [ **ACVR1B** ]  complex.  Ref: 7890768 J Biol Chem, 1995
- Activins, like other members of the TGF beta superfamily, initiate signaling by assembling a complex of two types of transmembrane serine threonine receptor kinases classified as type II ActRII  [ **ACVR2** ]  or ActRIIB and type I ALK4  [ **ACVR1B** ] .  Ref: 12665502 J Biol Chem< MedlineTA>J Biol Chem, 1995
- **ACVR1B**  ALK4  [ **ACVR1B** ] , activin receptor  [ **ACVR2** ]  type 1B gene mutations in pancreatic carcinoma.  Ref: 11248065 Proc Natl Acad Sci U S A, 2001
- This was accompanied by strong upregulation of the activin receptor  [ **ACVR2** ]  ALK 4  [ **ACVR1B** ]  ActR IB  [ **ACVR1B** ]  and redistribution of the TGF beta responsive ALK 5 TbetaR I.  Ref: 11472976 Am J Respir Cell Mol Biol, 2001
- A recently cloned inhibin binding protein, InhBP p120, associates strongly with the type IB activin receptor  [ **ACVR2** ]  Alk4  [ **ACVR1B** ]  in a ligand responsive manner and interacts to a lesser extent with other activin and bone morphogenetic protein BMP type I and activin type II receptors.  Ref: 11266516 Mol Endocrinol, 2001
- When coexpressed with type I activin receptor  [ **ACVR2** ]  ActR I or ActR IB  [ **ACVR1B** ]  in concert with type II receptor ActR II  [ **ACVR2** ] , Smad7 efficiently reduced an early transcriptional response mediated by ActR I but had only a minimal effect on the response mediated by ActR IB  [ **ACVR1B** ] .  Ref: 10828018 Blood, 2000
- Surprisingly, activin had no effect on follistatin mRNA levels, despite the fact that FS D1h cells express ActRII  [ **ACVR2** ] , ActRIIB, and ALK 4  [ **ACVR1B** ]  ActRIB.  Ref: 12538636 Endocrinology, 2003
- Overexpression of wild type activin receptor  [ **ACVR2** ]  alk4  [ **ACVR1B** ]  1 restores activin antiproliferative effects in human pituitary tumor cells.  Ref: 12364468 J Clin Endocrinol Metab, 2002
- PCR products with the expected sizes for ActR I, ActR IB  [ **ACVR1B** ] , ActR II  [ **ACVR2** ] , and ActR IIB mRNAs were detected in freshly dissociated and 5 day cultured granulosa luteal cells.  Ref: 10210281 Life Sci, 1999
- The localizations of serine threonine kinase receptor mRNA for the novel type I TGF beta and or activin receptor  [ **ACVR2** ]  named B1 rat, ALK 4  [ **ACVR1B** ]  mouse or ActR IB  [ **ACVR1B** ]  human were demonstrated by in situ hybridization.  Ref: 9013782 Brain Res Mol Brain Res, 1996
- Analysis in native tissues from 12 normal thyroids, 16 goiters, and 5 Graves thyroids demonstrated mRNA expression of the activin subunit betaA, TGFbeta receptor type I and II, activin receptors type I receptors alk2 and alk4  [ **ACVR1B** ] , and activin type II receptors actRII  [ **ACVR2** ]  and actRIIb.  Ref: 11069203 Horm Metab Res, 2000
- Using the ActR IB  [ **ACVR1B** ]  and ActR IIB receptor isoforms, we have investigated the mechanism of activin receptor  [ **ACVR2** ]  activation.  Ref: 8622651 Mol Cell Biol, 1996
- Activins exert their effects by inducing heteromeric complexes of either of two different type I receptors, ActR I or ActR IB  [ **ACVR1B** ] , and either of two type II receptors, ActR II  [ **ACVR2** ]  or ActR IIB.  Ref: 7577669 Mech Dev, 1995
- The major activin receptors expressed on HaCaT cells were activin type II receptor ActR II  [ **ACVR2** ]  and activin type IB receptor ActR IB  [ **ACVR1B** ] .  Ref: 9605406 Genes Cells, 1998
- These affinities are similar to those for binding to a chimeric receptor in which the ECD1 of CRFR2beta replaces the ECD of the type 1B activin receptor  [ **ACVR2** ]  ALK4  [ **ACVR1B** ] .  Ref: 12611895 J Biol Chem, 2003
- In this study, we found the expression of activin type I receptors ActR I and ActR IB  [ **ACVR1B** ]  and type II receptor ActR II  [ **ACVR2** ]  on human fetal lung fibroblasts HFL 1 by RT PCR and immunocytochemistry.  Ref: 11261590 Life Sci, 2000
- SB 431542 is a potent and specific inhibitor of transforming growth factor beta superfamily type I activin receptor  [ **ACVR2** ]  like kinase ALK receptors ALK4  [ **ACVR1B** ] , ALK5, and ALK7.  Ref: 12065756 Mol Pharmacol, 2002
